# Editorial

**Published:** 1856-10

**Authors:** 


### EDITORIAL DEPARTMENT.

### SPIRITUAL MEDICINE.

We find upon our table this morning the following, transmitted through the post-office by the author or some friend, viz: "A remedy for flooding after confinement. ℞. Take a lump of alum about the size of a hickory nut into a cup of sweet milk—let it curdle, then give one teaspoonful every hour until better. Sure remedy. MADAME FRANK."

Such doctors and doctresses as the one to whom we are indebted for the revelation of this *very new* remedy, are now to be found in every region; and everywhere meet with sufficient encouragement to sustain them.

Absurdities in medicine have ceased to awaken the suprise of the intelligent medical man, but the recognition of such monstrous folly and dangerous trifling by enlightened community, can never cease to astonish us. Our hopes have been that, to educate the people, to some extent at least, in the fundamental departments of the healing art, must tend to drive from amongst us all impostors, however high their apparent attainments, and much more the fools who pretend to no learning or philosophy, but to having spent many years among the Indians or Hottentots, and gathered all the medical knowledge of these very highly cultivated and refined nations, as the basis of practice.

But it is painful to observe that the patrons of all such tomfoolery, are often our educated and refined citizens, men whose common sense laugh at such pretension, while their credulity leads them, with the vulgar and ignorant, into the motley and hoodwinked throng that crowd the portals of ignorance and pretension.

It is strange to observe the extremes to which people run in such matters. At one time they will not be satisfied with anything but a constant gulping of bulky and nauseous nostrums; at another, feeding the fancy upon the imaginary contents of

9

globules, requiring the eye of faith to see them; now watching the habits of some animal to gather the medication which instinct indicates, again consulting "spirits of just men made perfect," to draw from the spirit land the celestial medicaments of its deceasless regions.

The uncertainties of our art, to some extent, are owing to circumstances beyond our control, and have given some ground for the ridicule which has long been heaped upon it. But how infinitely more numerous are those things which are the work of charlatans, empirics, fools and knaves which add pestice to the sneers of the community. A really very ludicrous idea must be formed of a department of knowledge claiming to be a science, if we survey the diverse systems of therapeutics around us, all claiming to be *the only true* application of the principles of medicine.

We see the Steamsonian Tom Doctor kindling his fires at the altar of Esculapius—and bringing about a welding heat, mends up the broken human machine—with his motto in glowing letters, "Heat is Life." The Hydropathist with his flood of water, pours Niagara into the steamer's Ætna, and paints in water colors "Cold is Life." The Anti-pathist perversely attempts to counteract disease by apposition therapeutics, while Homœpathy more amicably compromises the thing, by bringing about, substituting an artificial for a real disease.

Eclecticism proscribes all our heroic remedies, but retains all that are worthy of confidence—none which can do harm—professes to be a sort of *omnium gatherum* of all the good of all systems without the bad of any. The Mesmerizer, by various frictions and manipulations, paws his patient's disease out of him, while the Spiritualist brings down heavenly influences and celestial wisdom to sanctify his original and sure remedies, (such as the sample above.)

The Clairvoyant looks right through a body, has no more use for morbid anatomy or *post mortem* investigation, than their patients have for clothes to hide their nakedness from their penetrating gaze; sees every lesion, present, past and to come, and prescribes with a prescience only known to the gifted with this eyeless vision.

Is it not wonderful that folks should any longer die of disease? Out of so many infallible systems, strange is it not, that no one has yet drawn the "elixir vitæ." Alas, for all men die! aye, are killed by all, often.

It is because of the fallibility, nay, greater fallibility of all such systems, that regular medicine is still the dernier resort to the afflicted; when all *one idea* systems fail, then they turn, with shame, back to the system which offers all that can be hoped for in remedial aid.

They come with confessions, that that system which promises to do no more than *try* to cure disease upon such principles as learning, toil, investigation and philosophy have developed to us, and which is willing to own its impotency in many cases, and acknowledge their own error.

There are no specifics, yet all quackery builds itself upon the discovery of them, and enlightened communities follow every fool or knave who cries aloud "I have found it! I have found it!" People do not stop to inquire as to character of the pretender, but swallow, with eyes shut, the nostrum if it be such, or bow with blind credulity to silly manipulations or with awe-struck faith in supernatural revelations.

Our Saviour wept with bitter lamentations over a people and city who would not hear and believe the dictates of wisdom or words of mercy, though sanctioned by heavenly manifestations. He forebore with them, until the fullness of time brought the most calamitous judgments upon them. Men still will be deluded in spite of the lessons of reason and experience, and while we lament the disasters to which it constantly leads them, we have no resource but to continue to warn and labor to save them from impending physical ills, springing from their wayward credulity.

### PRESIDENT OF THE AMERICAN MEDICAL ASSOCIATION.

The mode, manner and place, where the above illustrious worthy shall be chosen, seems now to engage the attention of journalists. The *Buffalo Journal* has given vent to an accumulated *plethora*, which, it says, it endured until after the last meeting, because of personal considerations and motives, honorable and highly worthy of its able editor. We endorse every word contained in the article. This Society was never intended, nor should it be used, as a great *monster itinerant elevator* to be taken to certain localities, to elevate some one into conspicuity and prominence. Custom has sanctioned a practice, which, when scrutinised, will be undeistood as an arrangement by which, if a city opens its gates to its reception, with the hospitalities usually bestowed, the Association in return, is to honor such city with a President from one of its Faculty. This practice of running over this wide extended country, for the purpose of making celebrities of certain persons by constituting them officials and dignitaries, has an aspect of humility about it, and we think the dignity of that learned body, or the profession, is not materially improved thereby. Such would be the conclusion to which the impartial observer would come. But we do not particularly complain of the results thus far. Those who have been chosen heretofore to preside over the deliberations of that body, have, in the main, been men who have done honor to their calling, by the devotion exhibited during long years of patient toil to the profession and humanity, and, by their talents and researches, have contributed to the sum of scientific knowledge in the country. But there are yet those whose heads are grey in the service, who have all their lives devoted their best energies to the good of mankind, the profession and the advancement of science, who stand prominent, respected and honored by the profession and the world, but who can never reach to the honors of the first official position in the Association, simply because they do not happen to reside in a town or city large enough to promise the profuse hospitalities of some of the larger and more aristocratic cities. Besides this, it would *derogate too much from the dignity* of the Association, to hold its meetings in anything else than a *large* city.

But as custom has thus far sanctioned a procedure, which we had hoped the session in New York had broken up, we do not see how it can be changed at this time, nor do we wish to see any innovation upon past custom in our next Congress at Nashville, although he, upon whom the mantle *should* fall, wheresoever the next meeting be held, has, most magnanimously, waived the honors which custom would have given to him. This last, we are, by no means, in favor of, but the requisite steps can there be taken by an alteration in the organic law, to bring about a change in this regard. We would here give notice that, should the feature of itineracy of the Association be continued, we shall, in all due complacency, invite it once more over the Mississippi. Lest some of our eastern friends may smile at the proposition, we would say to them that, although the echo of the savage war-whoop has scarcely died away, yet here at the foot of the Rapids of the above mighty river, is a city of a population of fourteen thousand, whose citizens are as hospitable as those of any city of the Union. We mention this in advance that the members may be prepared to do us the honor of their presence—which is all we ask.

---

### COMMENCEMENT OF THE SESSION.

The opening exercises of the present session of the Medical Department of the University were very entertaining and instructive.

After prayer, by the Rev. Mr. Haines, of the M. E. Church, in which he, in a very feeling manner, implored the Divine blessing upon this and all other institutions established for education and improvement in scientific pursuits, Prof. Allen, Dean of the Faculty, introduced Prof. Marsh to the class and the audience, who proceeded to the delivery of an introductory, exhibiting the most marked ability on the part of its author, and acquirements of a rare and extensive range.

We are pleased to learn that the class have determined upon its publication, and its appearance is daily expected.

Although, then upon the very eve of one of the most exciting elections, which, in the history of the country, has been without parallel, yet there was a large class present, a majority of whom entitled to suffrage, yet anxious for scientific advancement, chose to surrender their great privilege and came forward promptly. Since then, the class has been steadily increasing and now numbers between seventy and eighty students, as promising in point of morals, intellect and acquirements as ever assembled in any school in the country.

Prof. Meeker, whose appointment to the chair of Anatomy, we noticed in a former issue, is delivering a most able, interesting and instructive course on his branch. The class, without exception, are enthusiastic in their expressions in his favor. He has the advantage of long experience as a teacher, and as an Anatomist and Surgeon, has no superior in the West. Unpretending and modest in his manners, plain and social in his feelings, of elevated sentiment and unblemished character, he has, and ever will secure, the esteem, confidence and affection of all those with whom he is brought into close association socially and professionally. Like some others we wot of, his profession has been, and is, the grand ruling and controlling motive in life. Hence his prominence and well earned success.

Prof. Johns, (late of Baltimore,) to whom was assigned the department of Physiology and Therapeutics, is proving himself eminently competent to the position. To a thorough knowledge of his subject, he unites a happy tact in imparting and impressing, what he wishes to teach, upon the mind of the student. Chaste and animated in his style, he fixes the attention of his auditory and gives full assurance of his real worth as a lecturer. Having had more than ordinary advantages, he has faithfully and well improved all, and as a consequence, ranks among the first as a ripe scholar and a scientific physician.

The above gentlemen are additions to the corps of last winter, with all whom the public are more or less acquainted.

We feel that there is real cause of pride in the growing influence and usefulness of our State Medical College, and we are assured that our friends will be pleased to know that the efforts of the Faculty are all directed to insuring the future permanency of the Institution, and it affords us equal pleasure to be enabled to announce this to them.

### RETRIBUTIVE JUSTICE.

We see enough in the world around us, in the conduct of those whose opportunities of moral culture have ever been deficient, to sorrow and grieve over. But when we witness gross immoralities and dishonest practices, by those whose early education has been good, and whose acquirements and education should enable them to perceive the most delicate distinctions between *right* and *wrong;* we confess that our faith in the moralizing influence of education is somewhat shaken, and that after all, there is a constitutional depravity which no moral influence nor education could subdue.

The unhappy fate of Dr. Ramsey, of Georgia, has led to these reflections. He edited a spirited little periodical, called the "*Blister and Critic*," published in Atalanta, the place of his residence. Meanwhile he was carrying on a system of fraud upon the government by forging certain documents, was arrested, thrown into prison, where, almost in the morning of his life, he cut short his career by his own hands.

As we have already intimated, Dr. R., was comparatively a young man, and a man of some considerable native talent. But the high crime for which he was arrested and with which he was charged, was not, we will venture to assert, his first delinquincy. We saw him at St. Louis, at the meeting of the A. M. A., where we thought he disgraced himself and degraded the profession by conduct ungentlemanly and highly reprehensible. And yet, we believe, had he kept aloof from evil associations and concomitant vices and evil practices, with his talents and acquirements, he would have made an ornament to society and a bright light in the profession. We grieve over a loss thus wilfully and determinedly brought about, and would gladly throw the veil of obscurity over his acts, but for the value of the example. In our profession, the temptations to err are very numerous and strong, and the first obliquity should be avoided. If care be not exercised, one step in wrong will be followed by another, until the individual becomes bold and reckless in his immoralities, and hardened in the perpetration of his evil practices, he loses all shame, and will boldly and unblushingly mingle in society and the world, when his own conscience upbraids him with the assurance that his crimes have forfeited his right to an association with the pure and the good.

Dr. Ramsey is deplored, but it should prove a warning to others.

### "MEDICAL WORLD."

This title startles us with its vast comprehensiveness, and our readers may justly wonder what is meant by the expansiveness of the caption. Nor can we speak definitely of the matter ourselves. It is something of which we have only heard, and yet, we have regarded ourselves as material atoms of this universal brotherhood of medicine.

We have not heard the mandates issued precedent to the birth of this medical mundane, but judging from the indications which have reached us through other media, the pronunciamento has gone forth "Let there be light," and we are looking, anxiously and expectantly, for a ray or two.

Until then, we must be content to say to our readers, that a medical journal, of *enlarged pretensions* and the most *liberal* views, has been projected and is being published in Boston. Its editor is J. V. C. Smith, M. D., by some called *Alphabet Smith*, because, we presume, of the liberal draught he has made upon it for initials, which we think, by the way, indicated some sense on the part of his parents, as *Smith* is equivalent to being *anonymous*. While we do not wish to become his biographer, we will, however, simply say that he once occupied the dignified position of Mayor of Boston, and in this we thought we saw a peculiar "fitness of things."

What, in a particular manner, concerns the medical profession is, that he *was* the editor of the "Boston Medical and Surgical Journal," a non-committal, every-where-and-no-where-kind of periodical; everybody's friend and nobody's enemy; a friend of every system and opposer of none. There was no discrimination exercised in the articles which appeared in its columns, the puerile efforts of brainless tyros often being prominent over those of scientific observers in its pages.

Such has been the general complaint of the medical public, against a journal in which many good things were recorded, but which were scandalised by being placed alongside those of the grossest empiricism. But he now throws off all reserve and comes out, body and soul, from Alpha to Omega, from beginning to end, an out and out advocate for all systems, regular and irregular, in his new periodical, and we take this opportunity to write him down as precisely on par with McClintock *et id genus omne.*

But fortunately, the journal solely under his care has been taken from his hands, and is now acceptably and ably conducted by his successors.

His sordid nature, however, like an evil genius, hawked him around and offered him to the highest bidder, and now he hopes, as the bargain has been struck and the preliminaries entered into, that he will soon be in receipt of the "thirty pieces of silver."

All his prominence and all his influence was imbibed by him as his only source of vital element from the profession whose forbearance he so long trespassed upon, and whose confidence he has betrayed. He has ever been the recipient of large favors, but never returned a *quid pro quo.*

That prominence and that influence (which he never deserved) he now lays down at the feet of the enemies of legitimate medicine, to be used by them as authority to sustain them in their attacks upon it. His former recognition by the members of the regular profession will give them a very effective weapon in their future crusades. They will trumpet his defection from Maine to California, and cry out that "one of the standard bearers of Allopathy has capitulated, and without terms."

Let him receive from the hands of the profession that justice which he merits, and perhaps we may have, in the future, fewer exhibitions of Arnold's treason among us. Let him have the full benefit of the remaining letters of the alphabet from which to select characters to write *traitor* after his cognomen of so wordy length, and his titles of so thundering sound.

---

### TRANSACTIONS OF THE AMERICAN MEDICAL ASSOCIATION.

We have received a copy of the above, per express, and, from a cursory glance, are pleased with its appearance.

But in looking over the list of permanent members we find an error repeated which *occurred* in the transactions of the previous year, and which we hoped would be corrected in the one before us. The senior editor of this journal is represented as residing at Dubuque, and we cannot divine how this error, which originated at St. Louis, was made. To us it is a matter of some moment, in consequence of the misdirection, by those who have no other guide than these transactions, of papers and documents, and we ask those who have the means of an early correction, to do so as an act of justice to us. Our address is *Keokuk* instead of Dubuque.

---

On Saturday, Nov. 30, Prof. Hughes operated for the Stone, in the College, before the class, removing a calculus of about four drachms weight and of the triple phosphate variety. The Stone was partially encysted just above the neck, and, of course, behind the pubis, rendering the catching of it exceedingly difficult. The position of the stone was worthy of note, because of its being so unusual and the seeming mechanical impossibility of its becoming fast there, as whenever the bladder was distended to any degree, its gravity would have a tendency to keep the calculus from centact with the surface to which it was attached. The patient was between sixty and seventy years of age—had been operated upon a little less than a year ago, when two large calculi were removed. The one removed on Saturday was doubtless then overlooked, as from its position it was possible to reach it only by means of the crooked forceps, passed directly upward behind the pubis.

The Dr. operated by the bilateral method, with the double lithatome catchet. He remarked to the class that he preferred, in every case which he had seen, this mode of operation, as being less liable to accidental wounding of the other organs in the region.

The patient, as we learn, is doing well. A full report of the case will be given hereafter.

---

☞ Dr. Goadby, of the "*Medical Independent*" of Detroit, Michigan, has prosecuted Dr. Zina Pitzer, President of the American Medical Association, for slander. We have not learned what were the offensive words spoken.

### OUR JOURNAL.

It is with regret that we have to announce the continued illness of the senior editor, Prof. McGugin, of the Journal. For several weeks he has been confined to his bed, unable to bear the upright position. Most of the editorial of the present number has been written by him while in bed, harrassed by pain and wearied with sleepless nights and restless days. Nor is it as yet possible to prognose respecting the time when he shall obtain relief from his suffering, and be again able to resume professional duty.

While this has been the case with the senior, unavoidable absence on the part of the junior editor, and, as we announced in our last, difficulties in obtaining compositors in the publishing office, have conspired to prevent the appearance of this and the preceding number at the proper time, and, as a consequence, the next, the closing number of the present volume, will be late in its issue.

To the editors, this is a source of anxiety and regret, greater, we doubt not, than to our readers and subscribers, for we not only love to be true to promises made, but are lovers of *punctuality* for itself. But to our subscribers we must say, we have at the same time, "somewhat against thee," and, also, that it is in part your fault that we are behind hand as to time. You will give us credit, we feel assured, for not belonging to that class of editors who are forever grumbling and growling at the remissness of their friends. We have striven to abstain from parading our necessities and your obligations before you, hoping and believing that, if we should but discharge to the full our duty, our subscribers would be prompt to fulfil theirs. Our faith is not "waxed cold" even yet, for we cannot doubt that those of the profession who have been the readers and *subscribers* of the *Journal*, have only failed to pay up their subscriptions from simple forgetfulness or ignorance of the fact that *their organ*, must depend, for a vigorous and healthy vitality, upon the *material aid* which it shall receive from its friends. Thus far we have labored in the work of publishing a journal which should be a creditable organ for the profession in this portion of our great commonwealth, not only without any requital for our services, or even the hope of it, but even at a large yearly outlay from our own hard earned means for its support We have looked forward to the time when the profession should fully esteem the importance of the work to the well being of medical science and the good of humanity, and believed that they would help us in bearing the burden. Year by year our subscription list has increased, and the press and numerous members of the medical fraternity have bestowed their encomiums upon the character and standing of our periodical, and yet it has failed to pay for itself, not because the subscription list is now too small, but because there is a large discrepancy between its footing up and the receipts of money.

Now, we appeal to you, to know if this is as it should be. We feel that the *Journal* is a necessity to the profession, not to us more than to all, and in view of the position which the *Iowa Medical Journal* holds in the ranks of journals over our land, we are free to express our pride, that, by the aid of the profession, we have been able to accomplish so much. And we ask of the subscribers of the *Journal*, that they promptly discharge their indebtednesss, in order that it may be freed from the debts under which it is struggling, and thereby free us from the embarrassments into which its publication has involved us.

We are anxious to commence another year free from any debt of the present year's contracting. Those which we have incurred and discharged in previous years, since the commencement of this undertaking are out ot the way, and we congratulate ourselves upon having been able to spare the means of so discharging them, satisfied that the money was well spent.

In the next number we shall send bills to all those in arrears, asking them to pay up, and, also, that they and all of our subscribers, will send on the price of subscription for the coming year. And may we not ask of you, our patrons, that you will, each and all of you, consider it a part ot your work in the profession to solicit new subscriptions, and thus assist to place your JOURNAL in a position of permanent pecuniary independence?

